# Enhanced neuro-ophthalmologic evaluation to support separation of craniopagus twins

**DOI:** 10.1093/jscr/rjaa606

**Published:** 2021-02-04

**Authors:** Sohaib R Rufai, Sri Gore, Sian E Handley, Oliver R Marmoy, Juling Ong, David J Dunaway, Noor ul Owase Jeelani

**Affiliations:** Clinical and Academic Department of Ophthalmology, Great Ormond Street Hospital for Children, London, UK; The University of Leicester Ulverscroft Eye Unit, Leicester, UK; UCL Great Ormond Street Institute of Child Health, London, UK & Craniofacial Unit, Great Ormond Street Hospital for Children, London, UK; Clinical and Academic Department of Ophthalmology, Great Ormond Street Hospital for Children, London, UK; Clinical and Academic Department of Ophthalmology, Great Ormond Street Hospital for Children, London, UK; Clinical and Academic Department of Ophthalmology, Great Ormond Street Hospital for Children, London, UK; UCL Great Ormond Street Institute of Child Health, London, UK & Craniofacial Unit, Great Ormond Street Hospital for Children, London, UK; UCL Great Ormond Street Institute of Child Health, London, UK & Craniofacial Unit, Great Ormond Street Hospital for Children, London, UK; UCL Great Ormond Street Institute of Child Health, London, UK & Craniofacial Unit, Great Ormond Street Hospital for Children, London, UK

## Abstract

Craniopagus conjoined twins are extraordinarily rare and present unique challenges to the multidisciplinary team. There is a paucity of literature on optimizing neuro-ophthalmologic evaluation in craniopagus twins. Herein, we present our enhanced neuro-ophthalmologic evaluation and management in 17-month-old male craniopagus twins, uniquely using handheld optical coherence tomography (OCT) plus portable slit-lamp biomicroscopy, indirect ophthalmoscopy and modified forced-choice preferential looking assessment. Staged surgical separation was supported by enhanced neuro-ophthalmologic evaluation, detailed radiology, three-dimensional printing and virtual reality simulation. This represents the fourth separation of craniopagus twins by our unit.

## INTRODUCTION

Craniopagus conjoined twins are extraordinarily rare, occurring in one in 2.5 million births and representing only 2–6% of conjoined twins [1, 2]. A recent systematic review [3] outlined factors for surgical success, but there is a paucity of literature on optimizing ophthalmologic evaluation in craniopagus twins where highly specialized methods are required. Given that such cases are very rarely encountered, it is important to share lessons learned from each case.

Craniopagus twins present unique challenges to the multidisciplinary team, including the ophthalmologist, due to their unique anatomy [4]. Visual assessment using forced-choice preferential looking cards is difficult to perform in the normal manner as the twins are unable to stand or sit upright. Table-mounted optical coherence tomography (OCT) devices and conventional slit-lamp biomicroscopes are unsuitable. Herein, we present our specialized and modified approach to neuro-ophthalmologic evaluation and management in a case of male craniopagus conjoined twins.

## CASE REPORT

**Figure 1:**
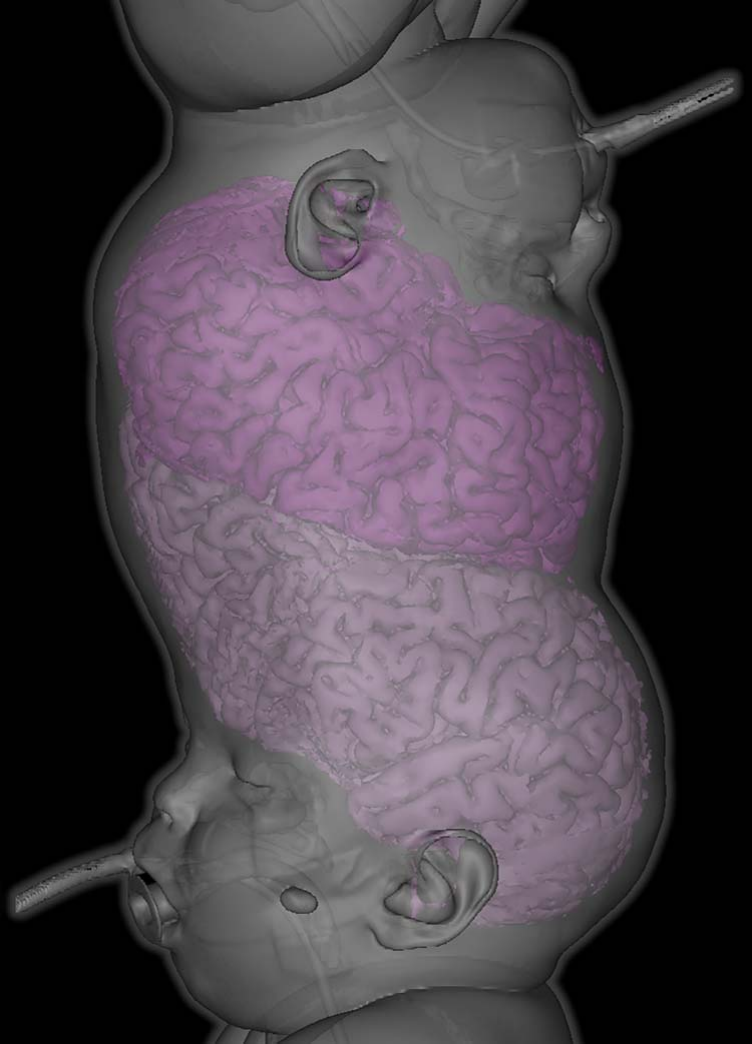
Three-dimensional reconstruction demonstrating total vertical (Type II) craniopagus malformation.

Male craniopagus conjoined twins were referred to our unit at 17 months old on 2 December 2019 from Antalya, Turkey. They were delivered at 32 weeks by caesarean section. We diagnosed total vertical craniopagus malformation, featuring continuous cranium and inter-twin axial facial rotation (Type II) [4], which can be appreciated on three-dimensional reconstruction (Fig. 1). There were no major ocular concerns reported by the parents.

**Figure 2:**
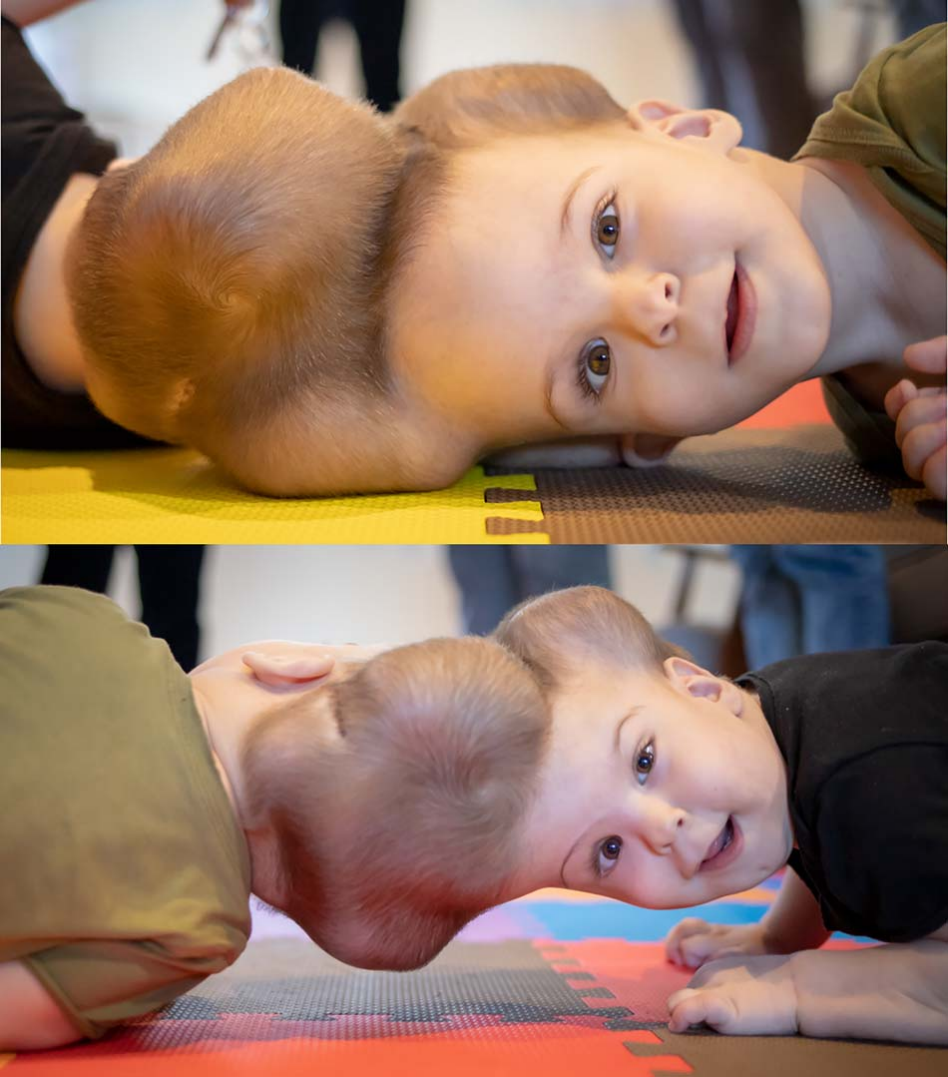
Tissue expansion performed to enable separation and craniofacial reconstruction.

**Figure 3:**
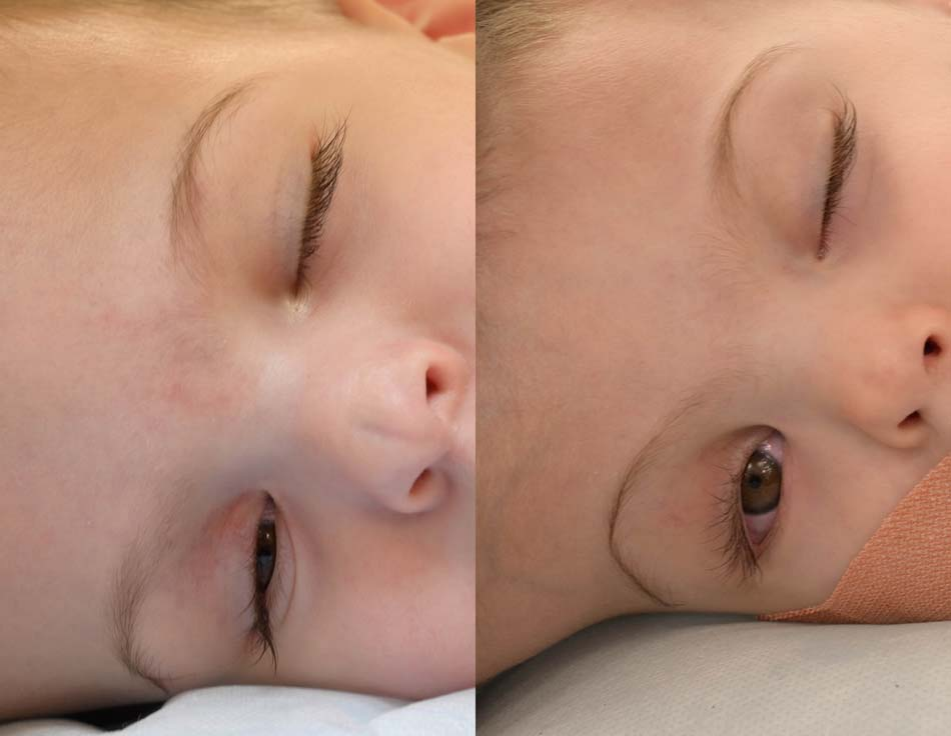
Lagophthalmos appreciated in right eye of both twins under general anaesthesia. Twin 1 (left) displayed right lagophthalmos of 3 mm whereas Twin 2 (right) displayed right lagophthalmos of 6 mm.

At initial ophthalmic examination, we noted that right brow position in each twin was abnormally high due to scalp shortage. Both twins were visually alert, fixing and following well, making eye contact, responding to smiling and demonstrating age appropriate behaviour. Binocular visual acuity (VA) was measured by forced-choice preferential looking using Keeler Acuity Cards (Keeler Ltd, Windsor, UK). VA fell within normal limits for corrected age in both twins (Twin 1: 6.4 cycles/degree; Twin 2: 4.7 cycles/degree) [5]. Due to the twins’ position, both lying on their right sides in bed, cards were held horizontally and vertical eye movements used as responses, then rotated vertically for confirmation of responses towards threshold. Eye movements were full. There was no relative afferent pupillary defect in either twin.

**Figure 4:**
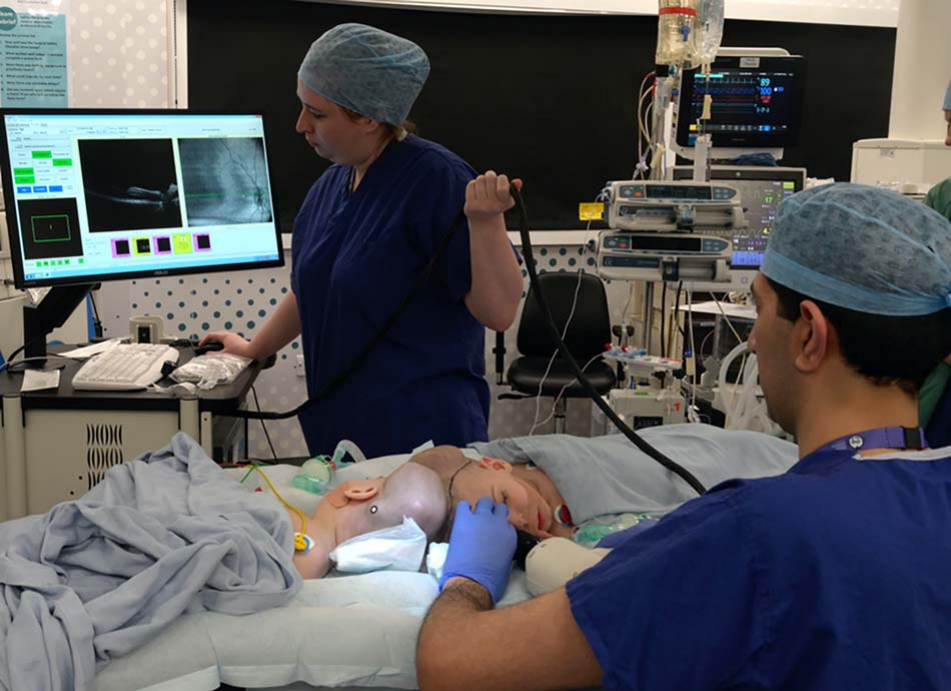
Handheld OCT acquisition. OCT image acquisition in lateral decubitus position under general anaesthesia using handheld OCT device.

In the first of four staged surgical separation procedures, we performed tissue expansion to permit separation and craniofacial reconstruction (Fig. 2). During this procedure, full anterior and posterior segment examination was achieved under general anesthesia. Examination findings for Twin 1 included right lagophthalmos of 3 mm (Fig. 3) due to abnormally high brow position causing right corneal punctate epithelial erosions, visualized using the Keeler Portable Slitlamp (Keeler Ltd, Windsor, UK). Anterior segment examination was otherwise normal. Indirect ophthalmoscopy revealed normal macular reflexes and healthy optic discs with cup-disc ratios of 0.3. Twin 2 had the same findings as Twin 1, except right brow was comparatively higher, causing 6 mm lagophthalmos (Fig. 3) and exposure keratopathy in the form of more widespread punctate epithelial erosions. Both were treated with chloramphenicol 1% eye ointment QDS and sodium hyaluronate 0.2% eye drops Q2H, to both eyes, resulting in clear corneas within 2 weeks.

**Figure 5:**
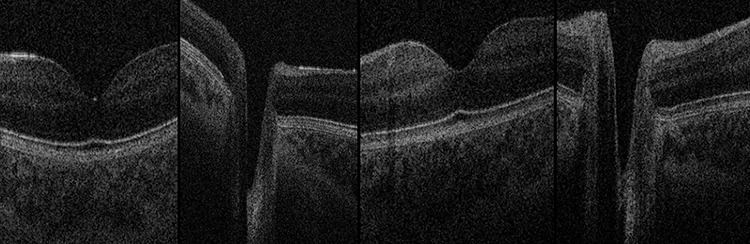
OCT Images. Normal foveae and optic nerve head appearance in both twins, excluding papilloedema. Far-left: Twin 1 normal fovea; centre-left: Twin 1 normal optic nerve head; centre-right: Twin 2 normal fovea; far-right: Twin 2 normal optic nerve head.

Handheld OCT was also performed using the Envisu C2300 (Leica Microsystems, Wetzlar, Germany) with each twin in the lateral decubitus position (Fig. 4), demonstrating healthy foveae and no optic nerve head swelling (Fig. 5). All OCT parameters fell within the normal range for age [6]. Our OCT acquisition protocol has been recently described [7].

**Figure 6:**
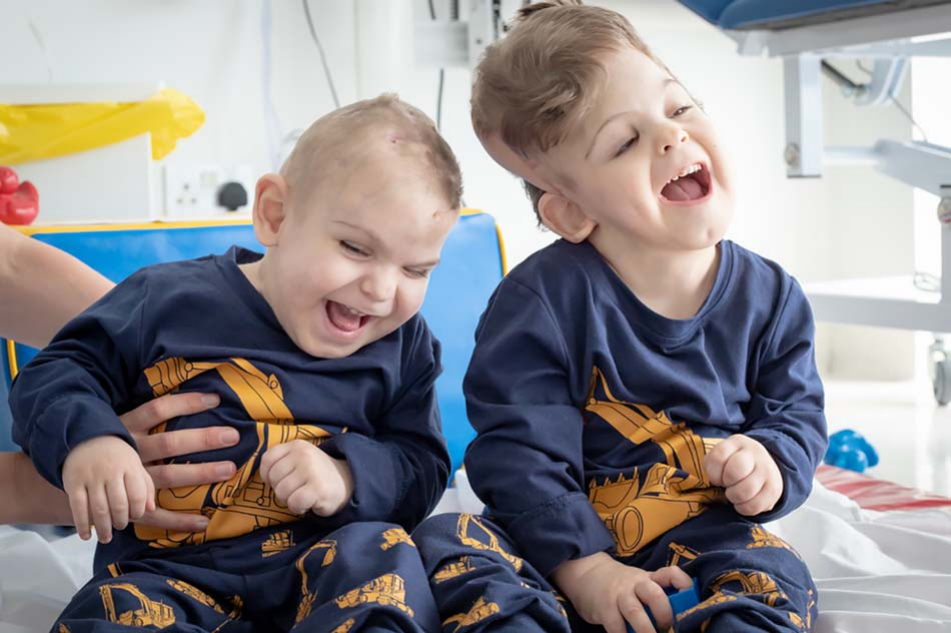
Twins post-separation. Twins post-separation taking part in physiotherapy.

Following four staged surgical procedures, the twins were successfully separated on 28 January 2020 and discharged from our unit on 9 June 2020 (Fig. 6). Brow positions grossly improved. VA reassessment was stable in Twin 2. VA reassessment was challenging in Twin 1, albeit he achieved 1.8 cycles/degree and OCT findings remained stable. Furthermore, on 10-month post-operative follow-up in Turkey, visual behaviour was reassessed by the senior surgeon (N.U.O.J.) and appeared grossly appropriate for age in both twins. Both require continued multidisciplinary care in Turkey. This represents the fourth successful separation of craniopagus twins at our unit using enhanced neuro-ophthalmologic evaluation, detailed radiology, three-dimensional printing and virtual reality simulation to support a staged surgical approach.

## DISCUSSION

Craniopagus twins require a highly specialized approach by the entire multidisciplinary team, including the ophthalmologist. Due to the rarity of craniopagus twins and paucity of literature on neuro-ophthalmologic evaluation, this experience provided several useful learning points. First, OCT image acquisition is possible in craniopagus twins. To our knowledge, this is the first published report of OCT examination performed in craniopagus twins, achieved using a handheld device. The OCT examination provided reassurance that foveal architecture and optic nerve head appearance was normal on baseline assessment, in addition to subsequently excluding retinal pathology or papilloedema in Twin 1 when visual behaviour appeared altered in the initial post-operative period. The portable slit-lamp enabled detailed anterior segment examination under general anesthesia, crucially revealing exposure keratopathy which was treated with intensive corneal lubrication. Our modified technique using forced-choice preferential looking cards enabled baseline VA assessment in both twins whilst conjoined.

This was the fourth successful separation of craniopagus twins at our unit. The surgical team adopted a staged approach with meticulous planning using detailed radiology, three-dimensional printing and virtual reality simulation.

In conclusion, a specialized and modified approach is required for detailed neuro-ophthalmologic evaluation and appropriate management in craniopagus twins. Portable slit-lamp biomicroscopy, indirect ophthalmoscopy and our modified technique using forced-choice preferential looking permitted detailed ophthalmologic evaluation, crucially detecting exposure keratopathy which was treated topically. Handheld OCT is possible and clinically valuable in craniopagus twins.

## PATIENT CONSENT

The patients’ legal guardian (father) consented to publication of this case report in writing. Both twins were consented and recruited onto our broader study, ‘Characterization of normal and abnormal ocular development using ultra-high resolution optical coherence tomography (UHR-SD OCT)’, for which ethics committee approval was granted by the East Midlands Nottingham 2 Research Ethics committee (UOL0348/IRAS 105137).

## FUNDING

The Medical Research Council (London, UK) (grant no.: MR/N004566/1). S.R.R. and S.E.H. are funded by National Institute for Health Research (NIHR) Doctoral Fellowship Awards. This work is supported by the NIHR Great Ormond Street Hospital Biomedical Research Centre. This case report presents independent research funded by the National Institute for Health Research (NIHR) and MRC. The views expressed are those of the author(s) and not necessarily those of the MRC, the NHS, the NIHR or the Department of Health and Social Care.

## CONFLICT OF INTEREST STATEMENT

The following authors have no financial disclosures: S.R.R., S.G., S.E.H., O.R.M., J.O., D.J.D. and N.U.O.J.

## AUTHORSHIP

All authors attest that they meet the current ICMJE criteria for Authorship.
